# Independent and Joint Associations of Lipoprotein(a) and Homocysteine with Carotid–Femoral Pulse-Wave Velocity in Adults with Treated Hypertension: A Single-Centre Cross-Sectional Study

**DOI:** 10.3390/jcm15155949

**Published:** 2026-07-30

**Authors:** Bibombe Patrice Mwipatayi, Reane Macaulay, Daniela Liezel Mwipatayi, Revathy Carnagarin, Ramon Lee Varcoe, Jacqueline Wong, Jonathan Golledge, Trevor Anthony Mori, Gerald Francis Watts, Markus Peter Schlaich

**Affiliations:** 1 School of Surgery, University of Western Australia, Perth, WA 6009, Australia; 2 Department of Vascular Surgery, Northern Hospital, Epping, VIC 3076, Australia; 3 Medical Department, Western General Hospital, Crewe Road South, Edinburgh EH4 2XU, UK; 4 Dobney Hypertension Centre, RPH Research Foundation Building, Research & Innovation Centre, 56 Murray Street, Perth, WA 6000, Australia; revathy.carnagarin@uwa.edu.au (R.C.); markus.schlaich@uwa.edu.au (M.P.S.); 5 Faculty of Medicine, University of New South Wales, Sydney, NSW 2052, Australia; r.varcoe@unsw.edu.au; 6 Department of Surgery, Prince of Wales Hospital, Randwick, NSW 2031, Australia; 7 Department of Vascular and Endovascular Surgery, Royal Perth Hospital, Perth, WA 6000, Australia; 8 Queensland Research Centre for Peripheral Vascular Disease, College of Medicine and Dentistry, James Cook University, 1 James Cook Drive, Douglas, Townsville, QLD 4811, Australia; 9 Australian Institute of Tropical Health and Medicine, James Cook University, 1 James Cook Drive, Douglas, Townsville, QLD 4811, Australia; 10 The Department of Vascular and Endovascular Surgery, Townsville University Hospital, Townsville, QLD 4814, Australia; 11 Medical School, University of Western Australia, Perth, WA 6009, Australiagerald.watts@uwa.edu.au (G.F.W.); 12 Cardiometabolic Service, Departments of Cardiology and Internal Medicine, Royal Perth Hospital, Perth, WA 6000, Australia; 13 Department of Nephrology, Royal Perth Hospital, Perth, WA 6000, Australia

**Keywords:** arterial stiffness, carotid–femoral pulse-wave velocity, homocysteine, hypertension, lipoprotein(a), peripheral artery disease

## Abstract

**Background/Objectives**: Carotid–femoral pulse-wave velocity (cfPWV) is the reference-standard non-invasive measure of central arterial stiffness and a validated marker of cardiovascular risk in hypertension. While lipoprotein(a) [Lp(a)] and homocysteine have each been implicated in vascular injury, their independent and combined associations with arterial stiffness in treated hypertension remain uncertain. In this study, we examined the associations of Lp(a), homocysteine, and peripheral artery disease (PAD) with cfPWV and assessed whether concomitant elevation of Lp(a) and homocysteine was associated with excess arterial stiffness. **Methods**: We conducted a single-centre, cross-sectional study of 366 consecutive adults with treated hypertension attending a tertiary hypertension centre and associated vascular service between February 2016 and October 2019. cfPWV was measured non-invasively through applanation tonometry using the SphygmoCor XCEL system. Multivariable robust linear regression was used to assess associations with cfPWV after adjustment for age, sex, body mass index, mean 24 h systolic blood pressure (BP), diabetes mellitus, chronic kidney disease, smoking status, low-density lipoprotein cholesterol, and PAD. Joint associations and interactions on multiplicative and additive scales were also evaluated. **Results**: Of 366 participants, 99 (27.0%) had high arterial stiffness, defined as cfPWV ≥ 10 m/s. After full adjustment, cfPWV increased by 0.13 m/s per 0.1 g/L increase in Lp(a) (95% CI, 0.08 to 0.18; *p* < 0.001) and by 0.26 m/s per 5 μmol/L increase in homocysteine (95% CI, 0.02 to 0.49; *p* = 0.03). Participants with concomitantly elevated Lp(a) and homocysteine had the highest adjusted cfPWV, with a mean difference of 1.56 m/s versus the low-Lp(a)/low-homocysteine group (95% CI, 1.00 to 2.13; *p* < 0.001). No meaningful multiplicative or additive interaction was observed. **Conclusions**: In adults with treated hypertension, higher Lp(a), higher homocysteine, and PAD were independently associated with greater central arterial stiffness. Concomitant elevation of Lp(a) and homocysteine identified participants with the highest cfPWV without evidence of synergistic interaction, supporting a complementary vascular-risk model.

## 1. Introduction

Arterial stiffness, commonly assessed using carotid–femoral pulse-wave velocity (cfPWV), is a robust marker of vascular ageing and an established indicator of cardiovascular risk [1,2]. In a systematic review and meta-analysis, higher aortic pulse-wave velocity was associated with an increased risk of future cardiovascular events and all-cause mortality [2]. In an individual-participant meta-analysis of prospective observational cohorts, aortic pulse-wave velocity predicted cardiovascular events and mortality beyond conventional cardiovascular risk factors and improved cardiovascular risk classification [3]. In adults with hypertension, a higher cfPWV reflects cumulative structural and functional injury to the arterial wall that is not fully captured by brachial blood pressure (BP) measurements alone [4,5]. Accordingly, cfPWV provides a clinically relevant intermediate vascular phenotype through which haemodynamic load, metabolic dysfunction, renal impairment, inflammation, and atherosclerotic disease may converge to influence cardiovascular risk [1,4,5].

Lipoprotein(a) [Lp(a)] is a genetically determined, low-density lipoprotein-like particle in which apolipoprotein(a) is covalently bound to apolipoprotein B100. Large prospective and genetic studies support Lp(a) as an important vascular-risk marker. In the Emerging Risk Factors Collaboration analysis of 126,634 participants from prospective studies, Lp(a) concentration was independently associated with coronary heart disease and ischaemic stroke after adjustment for conventional cardiovascular risk factors, whereas no clear association was observed with nonvascular mortality [6]. Contemporary consensus statements recognise Lp(a) as a causal risk factor for atherosclerotic cardiovascular disease and calcific aortic valve disease [7]. Elevated Lp(a) has also been associated with peripheral artery disease (PAD), abdominal aortic aneurysm, and adverse limb outcomes, supporting its relevance across multiple arterial territories [7,8]. However, the relationship between Lp(a) and arterial stiffness remains incompletely defined. In young adults with familial hypercholesterolaemia, van den Bosch and colleagues found no significant association between Lp(a) concentration and carotid pulse-wave velocity, suggesting that the vascular consequences of Lp(a) may not necessarily be expressed as early arterial stiffening in younger, intensively treated populations [9]. By contrast, in our companion analysis of the present hypertensive cohort, elevated Lp(a) and PAD were independently associated with higher cfPWV, without evidence that PAD modified the association between Lp(a) and arterial stiffness [10]. These apparently divergent findings suggest that the association between Lp(a) and arterial stiffness may depend on age, background vascular risk, PAD burden, and the clinical context in which Lp(a)-associated risk is expressed.

Homocysteine is a sulphur-containing amino acid generated during methionine metabolism and has long been implicated in vascular injury. Prospective observational evidence suggests that elevated homocysteine is associated with ischaemic heart disease and stroke risk, although the magnitude of association is modest and causality remains debated [11]. In a prospective-study meta-analysis, each 5 μmol/L higher homocysteine concentration was associated with higher all-cause mortality, cardiovascular mortality, and coronary heart disease mortality [12]. In hypertension cohorts, elevated homocysteine has been linked to vascular target-organ damage and arterial stiffness, supporting its relevance as a marker of adverse vascular phenotype [13,14,15]. Unlike Lp(a), which is predominantly genetically determined and structurally linked to an apolipoprotein B-containing lipoprotein particle, homocysteine is influenced by renal function, nutritional status, folate and B-vitamin metabolism, inflammation, and other metabolic factors [13,16,17]. Elevated homocysteine has been associated with endothelial dysfunction, oxidative stress, reduced nitric oxide bioavailability, vascular inflammation, and maladaptive extracellular matrix remodelling [17,18,19]. These mechanisms provide a plausible biological basis for an association between homocysteine and arterial stiffness, particularly in hypertension, in which renal dysfunction, vascular target-organ injury, and cumulative haemodynamic load frequently coexist.

Although Lp(a) and homocysteine arise from distinct biological pathways, both have been linked to atherosclerosis, thrombosis, endothelial injury, and adverse cardiovascular outcomes. Lp(a) may promote vascular injury through lipid deposition, oxidised phospholipid carriage, inflammation, and prothrombotic and antifibrinolytic effects, whereas homocysteine is more closely linked to endothelial dysfunction, oxidative stress, vascular inflammation, and impaired nitric oxide signalling [7,13,14,15,16,17,18,19]. Experimental and clinical data suggest that these pathways may intersect. Homocysteine has been shown to enhance the binding of Lp(a) to fibrin, and Foody and colleagues reported that the interaction between homocysteine and Lp(a) increased coronary artery disease risk in young men and women [20,21]. More recently, in a prospective cohort of patients with premature myocardial infarction, Wang and colleagues showed that concomitant elevation of Lp(a) and homocysteine was associated with the highest risk of major adverse cardiovascular events, with evidence supporting additive interaction [22]. These observations raise the possibility that the two biomarkers may jointly amplify vascular risk, though whether such combined effects extend beyond coronary-event risk to central arterial stiffness in treated hypertension remains uncertain.

In the present study, building on our previous analysis of Lp(a), PAD, and arterial stiffness in the same clinical cohort, we examined whether homocysteine provides incremental vascular-risk information beyond Lp(a), and whether concomitant elevation of Lp(a) and homocysteine is associated with greater central arterial stiffness than would be expected from their individual associations alone [10]. Because PAD is closely linked to Lp(a), systemic atherosclerotic burden, and cfPWV, we incorporated PAD as a key vascular covariate in the principal joint and interaction analyses [8,10]. We therefore assessed the independent and joint associations of Lp(a) and homocysteine with cfPWV in adults with treated hypertension, with particular attention to whether any combined biomarker effect persisted after accounting for clinically manifest PAD. We hypothesised that both biomarkers would be independently associated with higher cfPWV, and that their concomitant elevation would identify a higher-risk arterial stiffness phenotype characterised by complementary, rather than clearly synergistic, vascular-risk pathways.

## 2. Materials and Methods

### 2.1. Study Design and Population

We conducted a single-centre, cross-sectional study of consecutive adults with established treated hypertension who attended the Dobney Hypertension Centre and associated vascular and endovascular service at Royal Perth Hospital, Perth, Western Australia, between February 2016 and October 2019. The study period was specified before the analysis. Participants were eligible for inclusion if complete clinical, biochemical, medication, lipoprotein(a), homocysteine, and carotid–femoral pulse-wave velocity (cfPWV) data were available. The derivation of the analytical cohort is summarised in Supplementary Figure S1.

Because this was an observational analysis of a consecutive clinical cohort accrued over a predefined period, the analytical sample was determined by the number of eligible participants with complete data for the primary exposures, outcome, and prespecified covariates, rather than through a formal prospective sample-size calculation. The cohort was recruited from a tertiary hypertension centre and associated vascular service and was therefore enriched for patients with complex hypertension, vascular comorbidity, and higher cardiometabolic risk. This clinical profile is reflected in the prevalence of diabetes mellitus, PAD, chronic kidney disease, and multidrug antihypertensive therapy, including thiazide diuretics and spironolactone. Accordingly, the findings are most directly applicable to comparable specialist hypertension, renal, cardiometabolic, and vascular-risk settings, and should be extrapolated cautiously to unselected community-based hypertensive populations.

The study was conducted in accordance with the Declaration of Helsinki [23] and applicable International Council for Harmonisation Good Clinical Practice principles. Ethical approval was granted by the Royal Perth Hospital Human Research Ethics Committee (RGS0000006105). The committee approved a waiver of individual informed consent because the study was observational, non-interventional, and based on retrospective analysis of de-identified clinical data. Reporting followed the Strengthening the Reporting of Observational Studies in Epidemiology (STROBE) recommendations for cross-sectional studies [24], and the completed checklist is provided in Supplementary Table S1.

### 2.2. Clinical Assessment and Blood Pressure Measurement

All participants underwent a standardised clinical evaluation that included a comprehensive medical history, physical examination, assessment of cardiovascular risk factors and comorbidities, and anthropometric measurements. Clinical, biochemical, medication, comorbidity, ambulatory blood pressure (BP), and carotid–femoral pulse-wave velocity (cfPWV) data were extracted from structured clinical assessments, hospital medical records, laboratory records, ambulatory BP monitoring reports, and output files generated by the SphygmoCor XCEL system (AtCor Medical Pty Ltd., Sydney, Australia).

Extracted data were initially recorded in a password-protected Microsoft Excel spreadsheet (Microsoft Corporation, Redmond, WA, USA) and were subsequently transferred into a REDCap electronic database (Research Electronic Data Capture; Vanderbilt University, Nashville, TN, USA) for structured data management, consistency checking, and preparation of the analytical dataset [25,26]. The dataset used for statistical analysis was de-identified before analysis.

Ambulatory BP monitoring (ABPM) was performed using validated oscillometric devices, including Spacelabs monitors (Spacelabs Healthcare, Snoqualmie, WA, USA), Mobil-O-Graph monitors (IEM GmbH, Stolberg, Germany), and Oscar 2 ambulatory BP monitors (SunTech Medical, Morrisville, NC, USA). Recordings were obtained in accordance with international and European hypertension guidance [27,28,29]. BP measurements were scheduled every 15 min during the daytime period from 06:00 to 22:00 and every 30 min overnight from 22:00 to 06:00.

Mean 24 h systolic and diastolic BP values were derived from completed ambulatory recordings. Mean 24 h systolic BP was selected a priori as the principal ambulatory haemodynamic covariate because it provides an integrated measure of overall BP burden across the full recording period and is closely related to arterial stiffness. Daytime BP, nighttime BP, and nocturnal dipping status were not included as separate covariates in the primary multivariable models because of their correlation with mean 24 h BP and the risk of collinearity and over-adjustment.

### 2.3. Assessment of Arterial Stiffness

Arterial stiffness was assessed using carotid–femoral pulse-wave velocity (cfPWV), the reference-standard non-invasive measure of central arterial stiffness [1,4,5]. cfPWV was measured by applanation tonometry using the SphygmoCor XCEL system (AtCor Medical Pty Ltd., Sydney, Australia), in accordance with the manufacturer’s protocol and guideline-based recommendations for arterial stiffness measurement [4,5].

Measurements were performed by three trained operators experienced in the use of the SphygmoCor XCEL system. Because cfPWV acquisition by applanation tonometry is operator-dependent, measurement variability was minimised procedurally by using a single device platform, a standardised acquisition protocol, supine rest before measurement, and averaging of repeated technically acceptable measurements. Participants were studied in the supine position after at least 10 min of rest. Pulse waveforms were recorded from the carotid and femoral arterial sites, and cfPWV was calculated as path length divided by transit time. Two technically acceptable measurements obtained at trough were averaged for analysis.

For categorical analyses, high arterial stiffness was defined a priori as cfPWV ≥ 10 m/s, consistent with expert consensus recommendations for the clinical interpretation of increased aortic stiffness after standardisation of carotid–femoral distance measurement [5]. Higher cfPWV values indicate greater aortic stiffness and a more adverse arterial phenotype.

### 2.4. Laboratory Measurements, Exposures, and Covariates

Covariates were selected a priori based on clinical relevance and the published literature [1,3,4,5,6,7,8]. The principal adjustment set comprised age, sex, body mass index, mean 24 h systolic BP, diabetes mellitus, chronic kidney disease, smoking status, and low-density lipoprotein cholesterol. PAD was included as an additional vascular comorbidity because of its close clinical and biological relationship with Lp(a), systemic atherosclerotic burden, and arterial stiffness [7,8,30,31]. Recorded statin therapy was summarised in the baseline medication data and included in an additional sensitivity analysis to assess whether the associations of Lp(a), homocysteine, and PAD with cfPWV were materially altered by statin use.

The primary exposures were fasting plasma Lp(a) mass concentration, expressed in g/L, and plasma homocysteine concentration, expressed in μmol/L. Fasting blood samples were analysed by PathWest Laboratory Medicine Western Australia, Royal Perth Hospital, Perth, WA, Australia, using accredited clinical chemistry methods. Lp(a) was measured using an immunoassay-based method aligned with international recommendations for assay standardisation and traceability [32]. Additional laboratory variables included glycated haemoglobin, lipid profile, renal function measures, albumin–creatinine ratio, and high-sensitivity C-reactive protein.

Recorded statin therapy was defined using the composite medication variable in the study database and was further described according to individual statin agent where available. Statin therapy reflected routinely prescribed clinical medication rather than a study-administered intervention; therefore, no study drug manufacturer was applicable.

### 2.5. Definitions

Hypertension was defined according to the European Society of Cardiology/European Society of Hypertension and European Society of Hypertension guideline framework as an office systolic BP of at least 140 mm Hg, an office diastolic BP of at least 90 mm Hg, or current treatment with antihypertensive medication [28,29]. Participants receiving antihypertensive therapy were classified as having hypertension regardless of the office BP recorded at the study visit because treatment may have lowered measured BP below diagnostic thresholds.

Diabetes mellitus was defined according to established diagnostic criteria as a fasting plasma glucose concentration of at least 7.0 mmol/L, a glycated haemoglobin of at least 6.5%, and a 2 h plasma glucose concentration of at least 11.1 mmol/L during oral glucose tolerance testing or current treatment with glucose-lowering medication [33,34]. Participants receiving glucose-lowering therapy were classified as having diabetes mellitus regardless of contemporaneous glucose or glycated haemoglobin values.

Dyslipidaemia was defined as total cholesterol of 5.2 mmol/L or greater, low-density lipoprotein cholesterol of 3.4 mmol/L or greater, triglycerides of 1.7 mmol/L or greater, or current use of lipid-lowering therapy [35]. In participants at very high cardiovascular risk, a low-density lipoprotein cholesterol target below 1.8 mmol/L was considered optimal according to contemporary lipid management recommendations [35,36].

PAD was ascertained through a review of medical records and hospital coding data and was defined based on documented lower-extremity atherosclerotic disease. This included documented symptoms consistent with PAD, such as intermittent claudication, ischaemic rest pain, or tissue loss; abnormal lower-limb vascular assessment when available; and/or a history of lower-extremity revascularisation [30,31]. Systematic ankle–brachial index (ABI) measurement was not performed in all participants, and protocolised lower-limb duplex ultrasound was not undertaken in all borderline cases; this limitation is explicitly addressed in Section 4.

Stroke was defined as an acute focal neurological deficit attributable to cerebral infarction, intracerebral haemorrhage, or subarachnoid haemorrhage, and was confirmed by neuroimaging; transient ischaemic attacks were not included as stroke events [37,38].

Chronic kidney disease (CKD) was defined according to Kidney Disease Improving Global Outcomes (KDIGO) criteria as an estimated glomerular filtration rate < 60 mL/min/1.73 m^2^, albuminuria, or evidence of structural kidney abnormality persisting for at least 3 months [39].

### 2.6. Statistical Analysis

Continuous variables were summarised as mean ± standard deviation or as median with interquartile range, according to their distribution. Categorical variables were summarised as counts and percentages. Distributional assumptions were assessed through visual inspection of histograms and quantile–quantile plots, and with the Shapiro–Wilk test where relevant. Between-group comparisons were performed using Welch’s *t* test or the Wilcoxon rank-sum test for continuous variables and the chi-square or Fisher’s exact test for categorical variables, as appropriate.

Correlations between continuous variables were assessed using Spearman’s rank correlation coefficient, which was selected a priori because several key variables, including Lp(a), homocysteine, high-sensitivity C-reactive protein, triglycerides, the albumin–creatinine ratio, and cfPWV, were skewed and included extreme observations. Pearson’s correlation coefficient was considered but was not used for the primary correlation analyses because it assumes an approximately linear association between continuous variables and is more sensitive to outliers and leverage points. Correlation analyses were descriptive only; adjusted associations were evaluated using multivariable regression models.

The analytical strategy was designed to address two related questions: first, whether Lp(a) and homocysteine were independently associated with cfPWV, and second, whether their combined association differed from that expected based on their separate associations alone. The primary outcome, cfPWV, was analysed as a continuous dependent variable. Lp(a) and homocysteine were entered into the multivariable models on their original clinical scales and parameterised per 0.1 g/L and per 5 μmol/L, respectively. This preserved clinical interpretability and allowed effect estimates to be expressed in units meaningful to clinicians.

Mean 24 h systolic BP was selected a priori as the principal ambulatory haemodynamic covariate because it provides an integrated measure of overall systolic BP burden across the full recording period and is closely related to arterial stiffness. Daytime BP, nighttime BP, and nocturnal dipping status were not included as separate covariates in the primary multivariable models because these variables are derived from the same ambulatory BP recording, are closely correlated with mean 24 h BP, and could introduce collinearity or over-adjustment in a moderate-sized cohort.

For the primary multivariable analysis, robust linear regression was used to provide clinically interpretable effect estimates on the original cfPWV scale while reducing the influence of outlying observations. Robust regression was performed using Huber weighting with subsequent biweight iterations, as implemented in Stata’s rreg procedure [40]. The prespecified adjustment set included age, sex, body mass index, mean 24 h systolic BP, diabetes mellitus, chronic kidney disease, smoking status, and low-density lipoprotein cholesterol. PAD was then added in an extended model because of its close relationship with Lp(a), systemic atherosclerotic burden, and arterial stiffness.

Because cfPWV measurement through applanation tonometry may be operator-dependent, measurement variability was addressed procedurally rather than by operator-level statistical adjustment. Measurements were performed by three trained operators using a single device platform, a standardised SphygmoCor XCEL acquisition protocol, supine rest before measurement, and averaging of two technically acceptable measurements. Operator-level adjustment was not performed because operator identity was not consistently linked to each participant record, and repeated cross-operator measurements were not available.

The multiplicative interaction between Lp(a) and homocysteine was assessed by including a centred cross-product term in the PAD-adjusted model. Additive interaction was examined for continuous cfPWV using joint exposure categories and, for the binary outcome of high arterial stiffness, using Poisson regression with robust standard errors to estimate adjusted prevalence ratios [41]. For the binary outcome, additive interaction was quantified using the relative excess risk due to interaction, the attributable proportion due to interaction, and the synergy index, with bootstrap confidence intervals [42].

Receiver operating characteristic curve analysis was performed to assess the ability of Lp(a), homocysteine, and a combined Lp(a)–homocysteine–PAD model to discriminate high arterial stiffness, defined as cfPWV ≥ 10 m/s. Lp(a) and homocysteine were evaluated as continuous predictors, and discrimination was summarised using the area under the curve with 95% confidence intervals [43]. Data-derived optimal thresholds were identified using the Youden index [44], with corresponding sensitivity and specificity reported. These analyses were exploratory and were not intended to establish externally validated clinical decision thresholds.

Sensitivity analyses were performed to examine the robustness of the primary findings while preserving the clinical scale of the two primary exposures. Lp(a) and homocysteine were retained on their original scales in all sensitivity models. These analyses included ordinary least-squares regression with heteroscedasticity-consistent type 3 robust standard errors, median regression [45], additional adjustment for log-transformed high-sensitivity C-reactive protein, additional adjustment for recorded statin therapy, and additional adjustment for documented cardiovascular comorbidity. High-sensitivity C-reactive protein was log-transformed before inclusion because of its right-skewed distribution and because it was used as an inflammatory covariate rather than as a primary exposure.

Functional form was assessed using restricted cubic spline models, and model fit was compared using the Akaike information criterion, Bayesian information criterion, adjusted R^2^, and global nested-model comparison [46,47,48]. Additional exploratory analyses included a marginal structural model for high arterial stiffness and a Spearman correlation heatmap of key continuous variables.

Missing data were managed based on the analytical role of each variable. There were no missing values for the primary outcome, exposures, or covariates in the prespecified multivariable models. Consequently, both the primary and PAD-adjusted models were constructed using the complete cohort of 366 participants, which eliminated the need for imputation. In the descriptive analyses, any variables with incomplete data were reported along with their available denominators. In the exploratory sensitivity model that adjusted for documented cardiovascular comorbidities, instances of missing heart failure documentation (*n* = 91) were categorised as no documented heart failure. This classification was performed using deterministic coding, consistent with other comorbidities. While this adjustment accounted for documented conditions, it did not capture all unrecorded cases of heart failure. Therefore, the model included the full cohort (*n* = 366).

All tests were two-sided, and a *p* value below 0.05 was considered statistically significant. Because interaction, discrimination, and sensitivity analyses were exploratory, emphasis was placed on effect estimates, confidence intervals, consistency of findings, and clinical interpretation rather than on *p* values alone. Analyses were performed using Stata Statistical Software, version 17 (MP64 edition; StataCorp LLC, College Station, TX, USA).

## 3. Results

### 3.1. Study Population

A total of 366 adults with established treated hypertension were included in the analytical cohort. The derivation of the cohort is shown in Supplementary Figure S1. High arterial stiffness, defined a priori as cfPWV ≥ 10 m/s, was present in 99 participants (27.0%), whereas 267 participants (73.0%) had cfPWV < 10 m/s.

Baseline characteristics of the overall cohort are presented in Table 1. The cohort reflected the specialist tertiary referral setting, with a high burden of cardiometabolic and vascular risk, including diabetes mellitus, peripheral artery disease, cardiovascular comorbidity, renal dysfunction, and multidrug antihypertensive therapy. Variables with incomplete ascertainment, including aldosterone, renin, thyroid-stimulating hormone, heart-failure status, and spironolactone use, are reported with their available denominators.

Clinical, biochemical, haemodynamic, lipid, inflammatory, renal, medication, and cardiovascular comorbidity characteristics according to arterial stiffness status are shown in Table 2. Compared with participants with cfPWV < 10 m/s, those with cfPWV ≥ 10 m/s were older, had higher mean 24 h systolic BP, and had a substantially higher prevalence of peripheral artery disease and diabetes mellitus. They also had higher Lp(a), higher homocysteine, higher glycated haemoglobin, a lower estimated glomerular filtration rate, and a higher albumin–creatinine ratio. The largest absolute standardised mean differences were observed for peripheral artery disease, age, and mean 24 h systolic BP, indicating that established vascular disease, ageing, and cumulative systolic BP burden were the principal clinical features distinguishing participants with higher cfPWV.

To further describe the clinical phenotype associated with arterial stiffness, participants were stratified by arterial stiffness status using the prespecified cfPWV threshold of 10 m/s [5]. Table 2 presents the clinical, biochemical, haemodynamic, lipid, inflammatory, and renal characteristics of participants with lower arterial stiffness and higher arterial stiffness.

Participants with higher arterial stiffness differed significantly from those with lower arterial stiffness across several clinically relevant domains. Compared with participants with cfPWV < 10 m/s, those with cfPWV ≥ 10 m/s were older, had higher 24 h systolic BP, and had a markedly higher prevalence of PAD (all *p* < 0.001). Diabetes mellitus, coronary artery disease, prior stroke, and statin therapy were also more common in the higher-stiffness group. Biochemical differences were consistent with a higher vascular-risk profile, with higher Lp(a), homocysteine, and glycated haemoglobin, a lower estimated glomerular filtration rate, and a higher albumin–creatinine ratio. The largest absolute standardised mean differences were observed for PAD, age, 24 h systolic BP, diabetes mellitus, and statin therapy, indicating that established vascular disease, ageing, systolic BP burden, and treatment complexity were principal features distinguishing participants with higher cfPWV.

### 3.2. Correlation Between Biomarkers and Pulse-Wave Velocity

**Figure 1:**
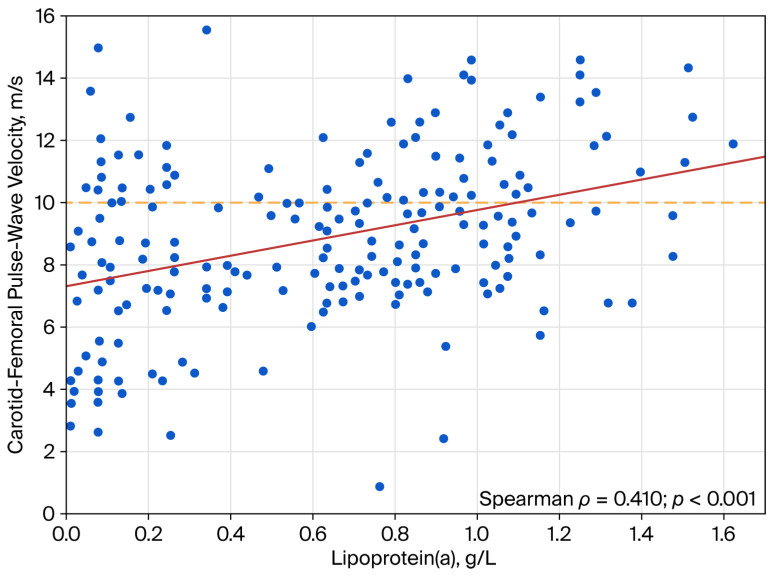
Association between lipoprotein(a) and carotid–femoral pulse-wave velocity. Scatter plot of Lp(a) concentration and cfPWV. The red line shows the fitted trend, and the orange dashed horizontal line marks the threshold for high arterial stiffness, defined as cfPWV ≥ 10 m/s. Spearman ρ = 0.410; *p* < 0.001. cfPWV, carotid–femoral pulse-wave velocity; Lp(a), lipoprotein(a).

**Figure 2:**
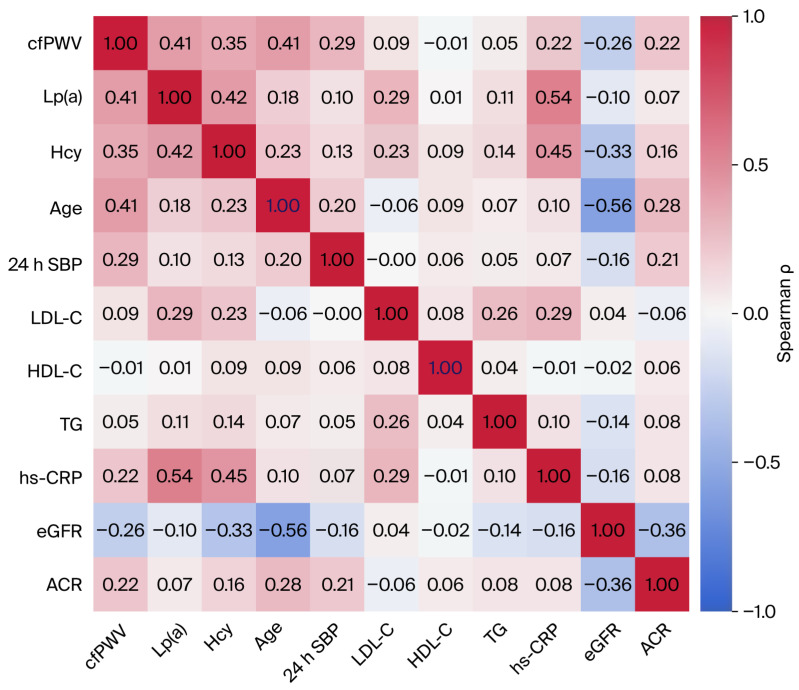
Spearman correlation heatmap. Pairwise Spearman correlations among continuous clinical, biochemical, haemodynamic, lipid, inflammatory, and renal variables. Red indicates positive correlations and blue inverse correlations. cfPWV, carotid–femoral pulse-wave velocity; Lp(a), lipoprotein(a); Hcy, homocysteine; SBP, systolic blood pressure; LDL-C, low-density lipoprotein cholesterol; HDL-C, high-density lipoprotein cholesterol; TG, triglycerides; hs-CRP, high-sensitivity C-reactive protein; eGFR, estimated glomerular filtration rate; ACR, albumin–creatinine ratio.

In unadjusted analyses, Spearman’s rank correlation was used to describe monotonic associations between biomarkers, haemodynamic variables, renal indices, inflammatory markers, and cfPWV. This approach was chosen because several variables were skewed and contained extreme values. Lp(a) showed a moderate positive correlation with cfPWV (Spearman ρ = 0.41; *p* < 0.001; Figure 1), and the magnitude of this association was similar to that observed for age (ρ = 0.41) and greater than that observed for 24 h systolic BP (ρ = 0.29), both of which are established determinants of arterial stiffness (Figure 2).

Homocysteine was also positively correlated with cfPWV (ρ = 0.35; Figure 2). In contrast, conventional lipid fractions showed weaker correlations with cfPWV, including LDL-C (ρ = 0.09), HDL-C (ρ = −0.01), and triglycerides (ρ = 0.05), suggesting that Lp(a) and homocysteine capture vascular-risk information not reflected by the standard lipid profile alone. Both biomarkers were positively correlated with high-sensitivity C-reactive protein (Lp(a): ρ = 0.54; homocysteine: ρ = 0.45), whereas homocysteine was inversely correlated with the estimated glomerular filtration rate (ρ = −0.33), consistent with the known influence of renal function on circulating homocysteine concentrations.

Lp(a) and homocysteine were moderately intercorrelated (ρ = 0.42; Figure 2), indicating related but non-redundant biomarker information. The magnitude of this correlation did not suggest problematic collinearity. These correlations were interpreted as descriptive unadjusted associations only and were not used as substitutes for adjusted regression modelling. Together, the unadjusted findings supported the inclusion of both biomarkers in subsequent multivariable models, alongside age, BP, renal function, systemic inflammation, and other relevant vascular covariates.

### 3.3. Multivariable Associations with Carotid–Femoral Pulse-Wave Velocity

In the primary multivariable robust linear regression model, higher Lp(a) and higher homocysteine were independently associated with higher cfPWV (Table 3). After adjustment for age, sex, body mass index, 24 h systolic blood pressure, diabetes mellitus, chronic kidney disease, smoking status, and low-density lipoprotein cholesterol, each 0.1 g/L increase in Lp(a) was associated with a 0.14 m/s higher cfPWV (95% confidence interval [CI], 0.08 to 0.19; *p* < 0.001), while each 5 μmol/L increase in homocysteine was associated with a 0.25 m/s higher cfPWV (95% CI, 0.01 to 0.49; *p* = 0.04).

After additional adjustment for PAD, the associations of Lp(a) and homocysteine with cfPWV were materially unchanged. Each 0.1 g/L increase in Lp(a) was associated with a 0.13 m/s higher cfPWV (95% CI, 0.08 to 0.18; *p* < 0.001), and each 5 μmol/L increase in homocysteine was associated with a 0.26 m/s higher cfPWV (95% CI, 0.02 to 0.49; *p* = 0.03). PAD was also independently associated with a higher cfPWV, with an adjusted difference of 0.75 m/s compared to participants without PAD (95% CI, 0.22 to 1.27; *p* = 0.006). These findings indicate that Lp(a), homocysteine, and clinically manifest lower-extremity atherosclerotic disease each provided independent information regarding arterial stiffness.

### 3.4. Discrimination of High Arterial Stiffness

**Figure 3:**
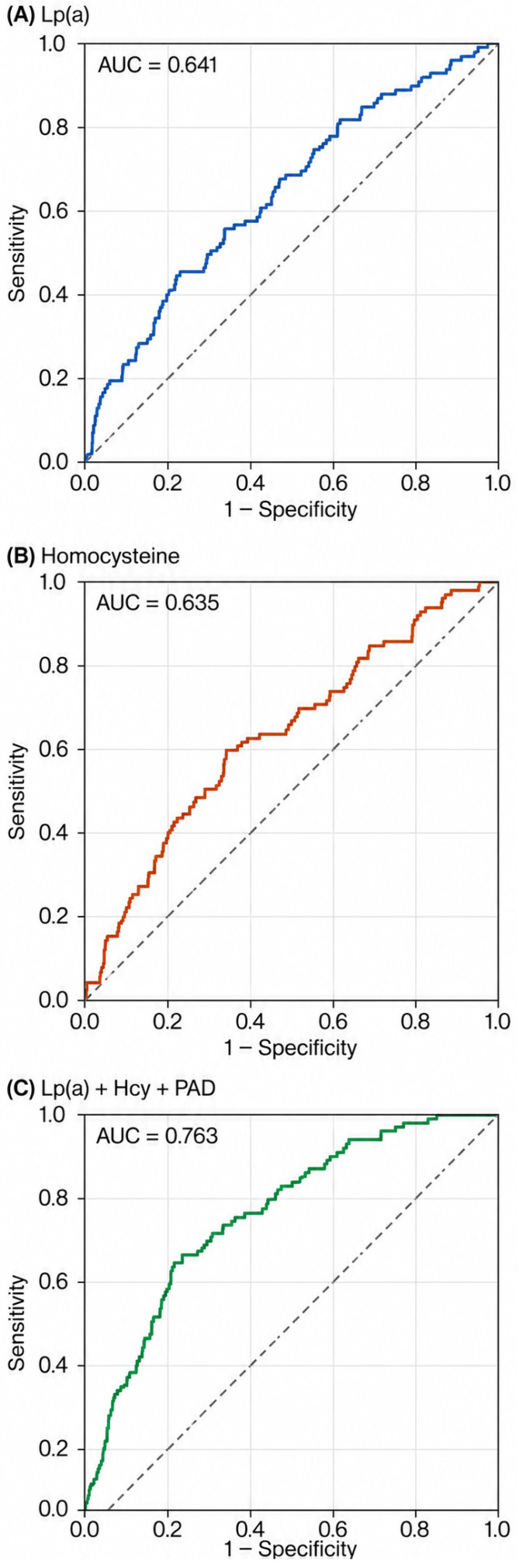
**Panel** (**A**) shows Lp(a), **Panel** (**B**) homocysteine, and **Panel** (**C**) the combined Lp(a)–homocysteine–PAD model. The grey dashed diagonal line indicates the line of no discrimination. AUCs were 0.641, 0.635, and 0.763, respectively. AUC, area under the curve; Hcy, homocysteine; Lp(a), lipoprotein(a); PAD, peripheral artery disease.

Receiver operating characteristic analysis showed modest discrimination for high arterial stiffness (defined as cfPWV ≥ 10 m/s) when either biomarker was considered alone (Figure 3). Lp(a) yielded an area under the curve of 0.641 (95% CI, 0.577 to 0.704; *p* < 0.001), with an optimal threshold of 0.77 g/L, a sensitivity of 49.5%, and a specificity of 74.2%. Homocysteine showed similar discrimination, with an area under the curve of 0.635, an optimal threshold of approximately 11.1 μmol/L, a sensitivity of 63.6%, and a specificity of 60.3%. A combined model incorporating Lp(a), homocysteine, and PAD improved discrimination, with an area under the curve of 0.763. In this combined model, PAD contributed the largest standardised coefficient, followed by Lp(a) and homocysteine.

### 3.5. Model-Predicted cfPWV Across the Joint Distribution of Lipoprotein(a) and Homocysteine

**Figure 4:**
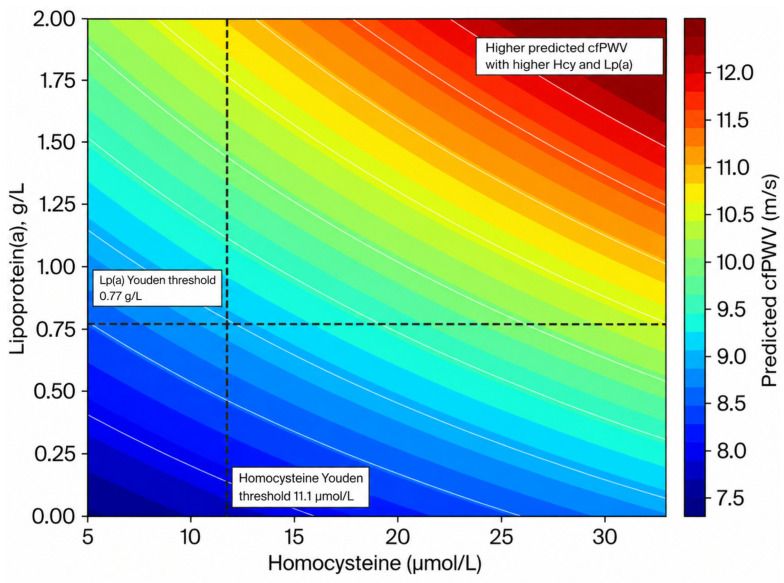
Predicted arterial stiffness across homocysteine and lipoprotein(a) concentrations. Contour plot of model-predicted cfPWV across homocysteine and Lp(a) concentrations. Warmer colours indicate higher predicted cfPWV, and contour lines indicate regions of similar predicted arterial stiffness. Dashed lines indicate the cohort-derived Youden-index thresholds for homocysteine (11.1 μmol/L) and Lp(a) (0.77 g/L). cfPWV, carotid–femoral pulse-wave velocity; Hcy, homocysteine; Lp(a), lipoprotein(a).

The joint association of homocysteine and Lp(a) with cfPWV was first examined across their continuous distributions. This model-based analysis was used to visualise how predicted central arterial stiffness varied across the combined biomarker range, rather than to display individual-participant observations. Predicted cfPWV increased gradually as homocysteine and Lp(a) concentrations increased, with the lowest predicted values observed when both biomarkers were low and the highest predicted values observed when both biomarkers were elevated. This gradient is shown in Figure 4, where warmer colours indicate a higher predicted cfPWV and white contour lines indicate regions with similar predicted arterial stiffness. The smooth contour pattern suggested a continuous increase in predicted cfPWV across the combined biomarker space, without an abrupt visual threshold.

To provide a clinically interpretable description of the observed cohort, participants were then classified using cohort-derived biomarker thresholds identified from receiver operating characteristic analysis for high arterial stiffness (Figure 3). The Youden index thresholds were >0.77 g/L for Lp(a) and >11.1 μmol/L for homocysteine. These cut points were used to define joint exposure categories for descriptive and interaction analyses and were not intended to represent externally validated diagnostic thresholds. Overall, 79 participants (21.6%) had concomitant elevation of both biomarkers. In this dual-elevation group, 48.1% had high arterial stiffness, and the mean cfPWV was 9.93 m/s. In comparison, among the 161 participants with both biomarkers at or below these thresholds, 16.1% had high arterial stiffness, and the mean cfPWV was 7.41 m/s. Thus, concomitant elevation of Lp(a) and homocysteine identified a subgroup with an approximately threefold higher prevalence of high arterial stiffness. Formal interaction analyses were subsequently performed to determine whether this observed joint pattern exceeded that expected from the individual biomarker associations.

### 3.6. Interaction Analyses

A centred Lp(a) × homocysteine cross-product term was added to the PAD-adjusted multivariable model to assess whether the association between Lp(a) and cfPWV varied according to homocysteine concentration. Both biomarkers were retained on their continuous clinical scales, and PAD was included in the model to account for the contribution of established peripheral atherosclerotic disease to arterial stiffness. There was no evidence of multiplicative interaction between Lp(a) and homocysteine (β_interaction, 0.01; 95% CI, −0.03 to 0.06; *p* = 0.55) (Table 3), indicating that the association between Lp(a) and cfPWV was not materially modified by homocysteine concentration.

**Figure 5:**
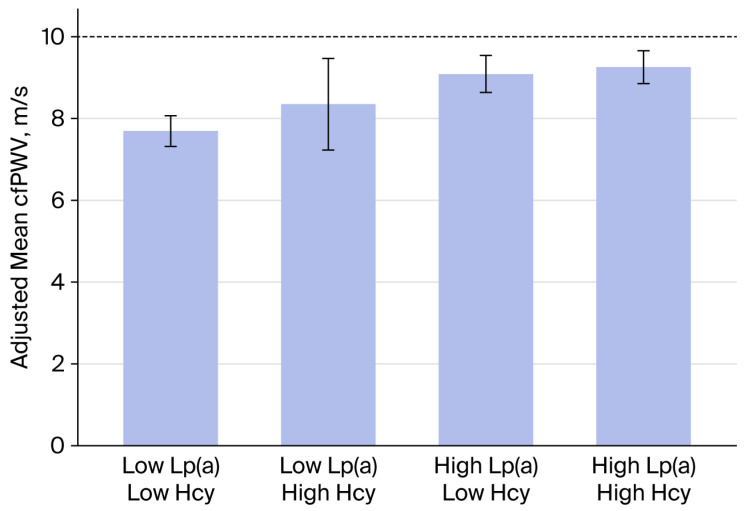
Adjusted carotid–femoral pulse-wave velocity according to joint biomarker status. Adjusted mean cfPWV across four Lp(a)–homocysteine groups. Error bars indicate 95% CIs, and the dashed line marks cfPWV ≥ 10 m/s. Models were adjusted for prespecified clinical covariates and PAD. CI, confidence interval; cfPWV, carotid–femoral pulse-wave velocity; Hcy, homocysteine; Lp(a), lipoprotein(a); PAD, peripheral artery disease.

To examine the joint clinical pattern, participants were also grouped into four threshold-defined Lp(a)–homocysteine categories. Adjusted mean cfPWV was highest among participants with concomitantly elevated Lp(a) and homocysteine (Figure 5). Compared with the low-Lp(a)/low-homocysteine reference group, the adjusted mean difference in cfPWV was 0.65 m/s in the low-Lp(a)/high-homocysteine group (95% CI, −0.55 to 1.86; *p* = 0.29), 1.39 m/s in the high-Lp(a)/low-homocysteine group (95% CI, 0.81 to 1.98; *p* < 0.001), and 1.56 m/s in the high-Lp(a)/high-homocysteine group (95% CI, 1.00 to 2.13; *p* < 0.001). Thus, concomitant elevation of Lp(a) and homocysteine identified participants with the highest adjusted cfPWV, though there was no evidence that the joint association exceeded that expected from the individual biomarker associations.

Formal interaction analyses provided no evidence that concomitant elevation of Lp(a) and homocysteine was associated with arterial stiffness beyond what was expected from their individual associations (Table 4). For continuous cfPWV, the additive interaction term was −0.49 m/s (95% CI, −1.85 to 0.87; *p* = 0.48). For high arterial stiffness, the relative excess risk due to interaction was −0.48 (95% CI, −1.72 to 0.45; *p* = 0.41), the attributable proportion due to interaction was −0.35 (95% CI, −1.37 to 0.30; *p* = 0.33), and the synergy index was 0.43 (95% CI, −1.14 to 2.61; *p* = 0.61). The marginal structural model interaction term was also non-significant (−0.20; 95% CI, −0.50 to 0.11; *p* = 0.20). Although several point estimates were below the null, the confidence intervals were wide and crossed the null value, providing no support for positive additive interaction or synergism. Consistent with these analyses, the observed-versus-expected prevalence comparison under an additive model showed that the prevalence of high arterial stiffness among participants with concomitantly elevated Lp(a) and homocysteine did not exceed that expected from the separate associations of each biomarker (Supplementary Figure S2).

### 3.7. Sensitivity Analyses

Sensitivity analyses were consistent with the primary findings (Supplementary Table S2). In the PAD-adjusted robust linear regression model, higher cfPWV remained associated with Lp(a), homocysteine, and PAD. The results were similar when ordinary least-squares regression was repeated using HC3 robust standard errors. Median regression confirmed the association for Lp(a), while the homocysteine estimate was directionally consistent but did not reach statistical significance. Additional adjustment for log-transformed high-sensitivity C-reactive protein did not materially alter the associations, and additional adjustment for recorded statin therapy also did not materially alter the associations of Lp(a), homocysteine, or PAD with cfPWV. In the statin-adjusted PAD model, Lp(a) remained associated with higher cfPWV (β, 0.126 m/s per 0.1 g/L; 95% CI, 0.074 to 0.177; *p* < 0.001), homocysteine remained associated with higher cfPWV (β, 0.249 m/s per 5 μmol/L; 95% CI, 0.009 to 0.488; *p* = 0.042), and PAD remained independently associated with higher cfPWV (β, 0.772 m/s; 95% CI, 0.239 to 1.305; *p* = 0.005). In a further exploratory model adjusted for documented cardiovascular comorbidity, Lp(a) remained independently associated with higher cfPWV (β, 0.128 m/s per 0.1 g/L; 95% CI, 0.076 to 0.179; *p* < 0.001), and PAD also remained associated with higher cfPWV (β, 0.713 m/s; 95% CI, 0.177 to 1.250; *p* = 0.009). The homocysteine estimate remained directionally consistent but did not reach conventional statistical significance (β, 0.230 m/s per 5 μmol/L; 95% CI, −0.013 to 0.473; *p* = 0.064). This documented cardiovascular comorbidity model included prior stroke, coronary artery disease, prior myocardial infarction, and documented heart failure. Restricted cubic spline modelling modestly improved model fit but did not change the direction or interpretation of the primary associations.

### 3.8. Restricted Cubic Spline Analyses

**Figure 6:**
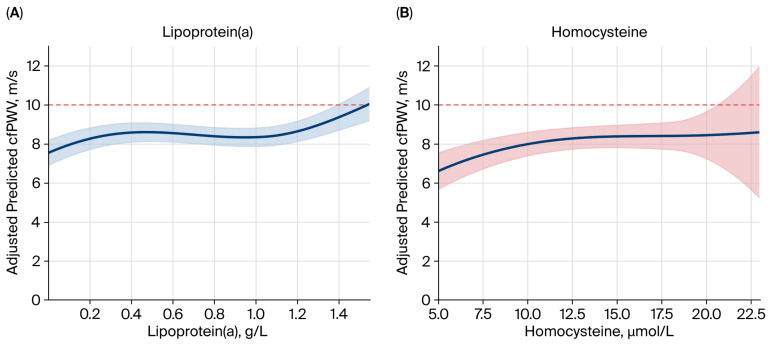
**Panel** (**A**) shows the adjusted restricted cubic spline association between lipoprotein(a) [Lp(a)] concentration and predicted carotid–femoral pulse-wave velocity (cfPWV). **Panel** (**B**) shows the adjusted restricted cubic spline association between homocysteine concentration and predicted cfPWV. In both panels, the solid navy line represents the fitted cfPWV estimate, the shaded band represents the 95% confidence interval, and the red dashed horizontal line marks the threshold for high arterial stiffness, defined as cfPWV = 10 m/s. Models were adjusted for prespecified clinical covariates, including peripheral artery disease. cfPWV, carotid–femoral pulse-wave velocity; Lp(a), lipoprotein(a).

Restricted cubic spline analyses further supported graded relationships between both biomarkers and predicted cfPWV (Figure 6). For Lp(a), predicted cfPWV increased from approximately 6.5 to 7.0 m/s at very low concentrations to more than 10 m/s at concentrations above approximately 1.3 g/L, reaching the clinically recognised threshold for high arterial stiffness at the upper end of the observed range. The association for homocysteine was more modest, increasing from approximately 7.6 m/s at 5 μmol/L to approximately 8.9 m/s at higher concentrations, with relative flattening thereafter. Although the spline model showed a modestly improved fit compared with the corresponding linear model, including a significant global comparison, the curves did not identify a discrete inflection point or clinically actionable threshold. Confidence intervals widened at the extremes of both biomarker distributions, consistent with lower data density in these ranges.

## 4. Discussion

In this cross-sectional study of adults with treated hypertension, higher Lp(a) and higher homocysteine were each independently associated with greater central arterial stiffness, as measured by cfPWV. These associations persisted after adjustment for established clinical and haemodynamic covariates and remained materially unchanged after additional adjustment for PAD. Although participants with concomitantly elevated Lp(a) and homocysteine had the highest adjusted cfPWV, formal interaction analyses did not show that their combined association exceeded that expected on either the multiplicative or additive scale. Taken together, these findings support a complementary vascular-risk model in which Lp(a), homocysteine, and PAD identify related but non-interchangeable domains of vascular injury associated with greater arterial stiffness.

The clinical relevance of these findings rests on the established prognostic importance of cfPWV. Central arterial stiffness is not simply a haemodynamic measurement; it reflects cumulative structural and functional injury to the aorta and large elastic arteries and is associated with cardiovascular events, all-cause mortality, and hypertension-mediated target-organ damage [1,2,3,4,5]. In the present cohort, 27.0% of participants met the prespecified threshold for high arterial stiffness. Participants with high cfPWV were older, had higher 24 h systolic BP, a greater prevalence of diabetes mellitus and PAD, higher Lp(a), higher homocysteine, greater statin use, and worse renal indices. This phenotype is consistent with the concept that arterial stiffness in treated hypertension reflects the cumulative burden of ageing, BP exposure, metabolic disease, renal dysfunction, treatment complexity, and systemic atherosclerotic injury rather than a single isolated risk pathway.

The present findings build on prior work in patients with hypertension by showing that homocysteine remained associated with cfPWV after adjustment for Lp(a), 24 h systolic BP, renal function, PAD, and conventional cardiovascular risk factors. In a previous analysis from the same hypertension clinical research setting, higher homocysteine was associated with hypertension-mediated vascular target-organ damage and was proposed as a marker of an adverse vascular phenotype [13]. The current study extends this observation to central arterial stiffness, a distinct vascular phenotype measured using cfPWV, and suggests that the association between homocysteine and arterial stiffness is not fully explained by Lp(a), PAD, renal dysfunction, or standard clinical risk factors. This distinction is biologically plausible because homocysteine and Lp(a) reflect different vascular-risk pathways. Homocysteine is a sulphur-containing amino acid generated during methionine metabolism, with circulating concentrations influenced by renal function, folate and B-vitamin status, nutritional factors, inflammation, and metabolic stress [13,16,17]. Higher homocysteine has been associated with increased arterial stiffness in clinical and population-based studies [14,15]. Mechanistic studies have linked homocysteine to endothelial injury and vascular inflammation [17], impaired nitric oxide bioavailability and endothelial nitric oxide synthase activity [18], oxidative stress and atherosclerotic vascular injury [17], and impaired endothelium-dependent vasorelaxation [19]. Taken together, these mechanisms provide a plausible biological basis for the observed association between homocysteine and cfPWV and support homocysteine as a complementary marker of arterial stiffness rather than a surrogate for Lp(a), PAD, renal dysfunction, or conventional cardiovascular risk.

The Lp(a) findings are also consistent with, and extend, our previous same-cohort Lp(a)–PAD analysis; in that study, elevated Lp(a) and PAD were independently associated with higher cfPWV, without evidence that PAD modified the association between Lp(a) and arterial stiffness [8]. The present study adds homocysteine to this framework and shows that Lp(a) remains independently associated with cfPWV after adjustment for homocysteine and PAD. This reinforces the interpretation that Lp(a) is a consistent correlate of arterial stiffness in this hypertensive cohort and suggests that the Lp(a) signal is not merely a surrogate for clinically manifest PAD, nor is it explained by homocysteine-related vascular injury.

The broader literature on PAD provides important context for these findings. Lp(a) is increasingly recognised as relevant not only to coronary artery disease and calcific aortic valve disease but also to PAD, adverse limb outcomes, and polyvascular risk [7,8]. In our narrative review of Lp(a) in PAD, we highlighted the biological plausibility of Lp(a)-mediated peripheral vascular injury, including atherogenesis, oxidised phospholipid carriage, inflammation, thrombosis, and impaired fibrinolysis [49]. Our subsequent systematic review and meta-analysis of elevated Lp(a) and outcomes after lower-extremity revascularisation further supported the clinical importance of Lp(a) in PAD, showing that elevated Lp(a) is associated with adverse limb outcomes after revascularisation [50]. Our present analysis complements this body of work by suggesting that, in patients with treated hypertension, Lp(a)-associated vascular risk is also reflected in central arterial stiffness. Thus, Lp(a) may reflect a vascular-risk domain that spans central arterial stiffening, PAD burden, and adverse limb prognosis.

The biological distinction between Lp(a) and homocysteine is central to the interpretation of this study. Lp(a) is an apolipoprotein B-containing lipoprotein particle in which apolipoprotein(a) is covalently linked to apolipoprotein B100. Its concentration is largely genetically determined, and its pathogenicity is thought to involve lipid deposition, oxidised phospholipid carriage, vascular inflammation, thrombosis, and antifibrinolytic effects [7,8,49]. Homocysteine, in contrast, is not a lipoprotein particle and does not primarily represent inherited lipid burden. Rather, it reflects amino acid metabolism and is influenced by renal, nutritional, metabolic, and inflammatory states. Its vascular effects are thought to be mediated through endothelial dysfunction, oxidative stress, nitric oxide depletion, smooth muscle proliferation, and matrix remodelling [13,14,15,16,17,18,19]. A previous mechanistic review by Tayal and colleagues emphasised that homocysteine and Lp(a) may contribute to atherosclerosis, but through partly distinct and partly overlapping pathways [51]. This conceptual distinction supports the present analytical approach, in which the two biomarkers were examined independently and jointly.

The possibility of an interaction between Lp(a) and homocysteine is biologically credible. Experimental data have shown that homocysteine can enhance the binding of Lp(a) to fibrin, providing a potential mechanistic link between hyperhomocysteinaemia, Lp(a), thrombosis, and impaired fibrinolysis [17]. Foody and colleagues have reported that homocysteine and Lp(a) interacted to increase coronary artery disease risk in young men and women [18]. More recently, in patients with premature myocardial infarction, concomitant elevation of Lp(a) and homocysteine was associated with the highest risk of major adverse cardiovascular events, with evidence supporting additive interaction [19]. These studies provide a strong rationale for testing whether combined Lp(a)–homocysteine exposure can highlight a more severe vascular phenotype in hypertension.

The present findings suggest that the interaction observed in coronary-event cohorts does not translate into a strong synergistic effect on central arterial stiffness in treated hypertension. Participants with co-elevation of Lp(a) and homocysteine had the highest adjusted cfPWV, but the increment beyond isolated high Lp(a) was modest. Similarly, neither the centred continuous interaction model nor the additive interaction measures supported a clear excess effect beyond the individual biomarker associations. This pattern is more consistent with the accumulation of vascular risk than with biological amplification. In practical terms, Lp(a) and homocysteine appear to provide complementary information: Lp(a) can be used to identify a genetically influenced lipid–thrombotic and atherosclerotic pathway, whereas homocysteine can be used to identify a metabolically and renally influenced endothelial and oxidative stress pathway.

Adjustment for PAD is an important feature of this analysis. PAD is a clinically manifest expression of systemic atherosclerosis and is strongly related to arterial stiffness, Lp(a), and adverse vascular outcomes [7,8,30,31,49,50]. Without accounting for PAD, the association between Lp(a), homocysteine, and cfPWV could partly reflect the burden of established lower-extremity atherosclerotic disease. In the present study, both biomarkers remained associated with cfPWV after adjustment for PAD, and the interaction analyses remained non-significant. These findings suggest that Lp(a) and homocysteine are not simply markers of existing PAD in this cohort but instead capture additional vascular-risk information relevant to central arterial stiffness.

Our receiver operating characteristic analyses should be interpreted cautiously. Lp(a) and homocysteine each showed only modest discrimination for high cfPWV when considered alone; this is not unexpected, because arterial stiffness is a complex vascular phenotype influenced by age, blood pressure, diabetes, renal function, inflammation, and established atherosclerotic disease. The combined model incorporating Lp(a), homocysteine, and PAD improved discrimination, with PAD contributing the largest standardised coefficient. This reinforces the clinical message that biomarkers should not be interpreted in isolation. Rather, their value may lie in complementing conventional clinical assessment and identifying biologically distinct components of residual vascular risk.

The sensitivity analyses supported the robustness of the primary findings without altering the clinical scale of either primary exposure. Lp(a) and homocysteine were retained on their original scales in all sensitivity models, preserving the clinical interpretation of the effect estimates. The direction and overall pattern of association remained similar when ordinary least-squares regression was repeated with heteroscedasticity-robust standard errors and when median regression was used. Additional adjustment for log-transformed high-sensitivity C-reactive protein did not materially alter the associations, while additional adjustment for recorded statin therapy did not explain the primary associations. Additional adjustment for available cardiovascular comorbidity showed that the Lp(a) and PAD associations remained robust, whereas the homocysteine estimate was attenuated and should therefore be interpreted more cautiously in relation to coexisting cardiovascular disease.

These findings have potential clinical implications, though they should not be overinterpreted. The data suggest that Lp(a), homocysteine, and PAD identify complementary features of an adverse vascular phenotype in patients with treated hypertension. This may be useful for refining vascular-risk characterisation, particularly in patients with persistent vascular risk despite treatment of conventional risk factors. However, this study does not establish temporality, does not prove causality, and does not show that modifying Lp(a) or homocysteine would reduce arterial stiffness. The findings should therefore be regarded as hypothesis-generating, and prospective validation is required to determine whether combined biomarker assessment improves clinical risk prediction beyond established clinical variables.

### 4.1. Clinical Implications and Implementation of cfPWV

cfPWV is the reference-standard non-invasive measure of central arterial stiffness and has established prognostic relevance in hypertension. Its clinical value lies in its ability to capture cumulative vascular injury that is not fully reflected by brachial BP, lipid concentrations, glycaemic status, or renal indices alone. In the present study, cfPWV provided a clinically interpretable vascular phenotype through which the associations of Lp(a), homocysteine, and PAD could be examined.

Despite this prognostic value, routine implementation of cfPWV in clinical practice remains challenging. Panayiotou and colleagues have highlighted limitations in the implementation of vascular ageing assessment in routine clinical practice, including practical barriers that restrict uptake outside specialised centres [52]. These barriers include the availability of validated devices, operator training, acquisition time, standardisation of measurement techniques, workflow integration, reimbursement, and uncertainty about how cfPWV should be incorporated into treatment decisions. These considerations are directly relevant to the present study, which was performed in a tertiary hypertension and vascular setting with access to trained operators and a standardised SphygmoCor XCEL protocol.

Accordingly, our findings should not be interpreted as supporting universal cfPWV screening in all patients with hypertension at this stage. Rather, they suggest that cfPWV may be most useful in specialist settings for refining vascular-risk assessment in patients with complex hypertension, PAD, renal dysfunction, elevated Lp(a), or elevated homocysteine. Wider implementation will require pragmatic studies showing that cfPWV measurement improves risk classification, changes clinical management, and leads to better cardiovascular or limb outcomes.

### 4.2. Strengths and Limitations

This study has several strengths: It examined Lp(a), homocysteine, PAD, and cfPWV in a well-characterised tertiary hypertension cohort with detailed clinical, biochemical, ambulatory BP, renal, lipid, inflammatory, medication, and vascular data. The analyses used clinically interpretable biomarker scaling, prespecified multivariable adjustment, PAD-adjusted models, multiplicative and additive interaction testing, and several sensitivity analyses, including robust regression, HC3 standard errors, median regression, inflammatory adjustment, statin adjustment, and adjustment for documented cardiovascular comorbidity.

Several limitations should also be acknowledged. First, the cross-sectional design precludes an assessment of temporality, as we cannot determine whether elevated Lp(a) or homocysteine preceded the development of greater arterial stiffness, accompanied it, or reflected established vascular injury. The findings should therefore be interpreted as associative and hypothesis-generating rather than causal.

Second, the study was based on a consecutive clinical cohort accrued over a predefined period rather than on a prospectively powered sample-size calculation. Although the analytical cohort provided sufficient precision for the principal multivariable associations, precision was more limited for subgroup, interaction, and sensitivity analyses. These analyses should therefore be interpreted cautiously and confirmed in larger prospective cohorts.

Third, the cohort was recruited from a tertiary hypertension centre and associated vascular service. This enabled detailed vascular phenotyping but enriched the cohort for complex hypertension, PAD, renal dysfunction, diabetes mellitus, cardiovascular comorbidity, and multidrug antihypertensive therapy. The findings are therefore most applicable to comparable specialist hypertension, renal, cardiometabolic, and vascular-risk settings, and should not be directly extrapolated to unselected community-based hypertensive populations.

Fourth, PAD was ascertained from clinical records, hospital coding data, available vascular assessments, and prior lower-extremity revascularisation rather than by systematic ABI measurement in all participants. Asymptomatic or subclinical PAD may therefore have been underestimated. Systematic ABI assessment, with lower-limb duplex ultrasound for borderline or abnormal findings, would have provided more accurate PAD classification and more precise adjustment for PAD-related confounding.

Medication and comorbidity data were also limited by the retrospective design. Antihypertensive medication classes were recorded, but dose, duration, adherence, treatment indication, and longitudinal treatment changes were not uniformly available. Analyses of individual antihypertensive drug effects would therefore have been vulnerable to confounding by indication. Similarly, while statin therapy was recorded and included in the sensitivity analysis, statin dose, intensity, adherence, duration, and treatment changes were not consistently captured. Residual confounding related to lipid-lowering therapy, including potential pleiotropic statin effects on endothelial function and arterial stiffness, cannot be excluded.

Heart-failure status was incompletely recorded. In the exploratory documented cardiovascular comorbidity-adjusted sensitivity model, missing heart-failure documentation was coded as no documented heart failure to preserve the full analytical cohort. This deterministic documented-disease coding avoided restriction to a selected complete-case subgroup, but residual confounding by unrecorded heart failure cannot be excluded.

cfPWV measurement using applanation tonometry is operator dependent. In this study, measurements were performed by three trained operators using a standardised SphygmoCor XCEL protocol, and two technically acceptable measurements were averaged to reduce random measurement variability. However, operator identity was not consistently linked to participant records, and repeated cross-operator measurements were not available. Formal operator-adjusted modelling or estimation of inter-operator reliability could therefore not be performed. In addition, cfPWV was assessed at a single study visit; repeated longitudinal measurements would have allowed better distinction between persistent arterial stiffness and short-term measurement variability and would have enabled assessment of arterial stiffness progression over time.

Finally, the study did not include direct measures of oxidised phospholipids, fibrinolysis, endothelial function, reactive oxygen species, interleukin-6, tumour necrosis factor-α, or other inflammatory and thrombotic biomarkers. Although high-sensitivity C-reactive protein was examined in the sensitivity analysis, it cannot fully capture the inflammatory, oxidative, prothrombotic, and endothelial pathways through which Lp(a) and homocysteine may influence vascular biology. Detailed ambulatory BP phenotypes, including daytime BP, nighttime BP, nocturnal dipping status, and BP variability, were also not modelled separately and should be examined in future studies.

### 4.3. Future Perspectives

The present findings should be regarded as hypothesis-generating and require confirmation in prospective studies. Future research should include longitudinal assessment of Lp(a), homocysteine, cfPWV, renal function, inflammatory markers, and vascular outcomes to determine whether these biomarkers predict progression of arterial stiffness over time. Systematic ABI measurement, with lower-limb duplex ultrasound where clinically indicated, would allow more accurate PAD phenotyping and more precise assessment of the relationship between peripheral atherosclerotic burden and central arterial stiffness.

Future studies should also include ethnically diverse populations, as Lp(a) concentrations vary substantially across ancestral groups. Detailed information on lipid-lowering therapy, statin intensity, antihypertensive treatment, folate and B-vitamin status, renal function, diet, and supplement use should be captured to reduce residual confounding, particularly for homocysteine. Genetically informed analyses may be especially useful for clarifying the causal contribution of Lp(a), whereas interventional studies will be required to determine whether lowering Lp(a), modifying homocysteine-related pathways, or targeting inflammatory and endothelial dysfunction can alter cfPWV or downstream cardiovascular and limb outcomes.

In clinical terms, these data support a complementary-risk model in which Lp(a), homocysteine, and PAD identify related but non-identical vascular-risk domains in treated hypertension. Whether combined biomarker assessment improves risk stratification beyond conventional clinical variables and whether this information should guide treatment intensity require further prospective validation.

## 5. Conclusions

Among adults with treated hypertension, higher Lp(a), higher homocysteine, and PAD were independently associated with greater central arterial stiffness. While participants with co-elevation of Lp(a) and homocysteine had higher cfPWV, the combined association did not exceed that expected from the individual biomarker associations on either the multiplicative or additive scale after adjustment for PAD. These findings support a complementary vascular-risk model rather than a clearly synergistic biomarker phenotype. Longitudinal and genetically informed studies are needed to determine whether these biomarkers improve risk stratification and whether modification of these pathways can alter arterial stiffness or downstream cardiovascular outcomes.

## Figures and Tables

**Table 1:** Baseline characteristics of the study population.

Characteristic	Overall Cohort (*n* = 366)
Age, y	56.3 ± 17.1
Male sex	206 (56.3)
Current smoking	193 (52.7)
Height, cm	169.6 ± 10.0
Weight, kg	84.2 ± 17.3
Body mass index, kg/m^2^	29.2 ± 5.4
Average 24 h systolic BP, mm Hg	135.9 ± 17.6
Average 24 h diastolic BP, mm Hg	77.7 ± 12.1
cfPWV, m/s ^†^	8.4 (6.9–10.1)
Diabetes mellitus	177 (48.4)
Peripheral artery disease	98 (26.8)
Coronary artery disease	57 (15.6)
Prior myocardial infarction	18 (4.9)
Prior stroke	24 (6.6)
Previous haemorrhagic stroke	10 (2.7)
Previous ischaemic stroke	14 (3.8)
Transient ischaemic attack	7 (1.9)
Heart failure	11/275 (4.0)
Lp(a), g/L ^†^	0.305 (0.080–0.868)
Homocysteine, μmol/L ^†^	11.0 (8.0–16.0)
High-sensitivity C-reactive protein, mg/L ^†^	3.00 (0.90–8.00)
Glycated haemoglobin, % ^†^	5.6 (5.1–6.5)
Dyslipidaemia	49 (13.4)
Low-density lipoprotein cholesterol, mmol/L	2.9 ± 1.2
High-density lipoprotein cholesterol, mmol/L	1.6 ± 1.0
Total cholesterol, mmol/L	4.9 ± 1.7
Triglycerides, mmol/L ^†^	0.39 (0.22–0.64)
Total cholesterol–high-density lipoprotein ratio	3.5 ± 1.4
Statin therapy	112 (30.6)
Rosuvastatin	56 (15.3)
Atorvastatin	46 (12.6)
Simvastatin	9 (2.5)
Pravastatin	1 (0.3)
Aldosterone, pmol/L ^††^	268.5 (197.2–388.5)
Renin, mU/L ^††^	22.5 (8.1–51.2)
Thyroid-stimulating hormone, mU/L ^††^	1.2 (1.0–2.0)
Estimated glomerular filtration rate, mL/min/1.73 m^2^	78.9 ± 17.3
Albumin–creatinine ratio, mg/mmol ^††^	2.85 (1.00–11.00)
Calcium channel blocker use	193 (52.7)
Angiotensin-converting enzyme inhibitor use	70 (19.1)
Angiotensin receptor blocker use	178 (48.6)
Beta-blocker use	124 (33.9)
Loop diuretic use	15 (4.1)
Spironolactone use ^††^	52 (15.9)
Thiazide diuretic use	115 (31.4)

Data are shown as *n* (%), mean ± SD, or median (IQR) for variables marked ^†^. Denominators varied for aldosterone (N = 226), renin (N = 228), thyroid-stimulating hormone (N = 229), heart failure (N = 275), and spironolactone use (N = 327) because of missing data, and percentages for these variables ^††^ were calculated with the available denominator rather than the overall cohort. Prior stroke excludes transient ischaemic attack. BP denotes blood pressure, cfPWV carotid–femoral pulse-wave velocity, and Lp(a) lipoprotein(a).

**Table 2:** Clinical, biochemical, and haemodynamic characteristics according to lower versus higher arterial stiffness.

Characteristic	cfPWV < 10 m/s (*n* = 267)	cfPWV ≥ 10 m/s (*n* = 99)	*p* Value	Absolute SMD
Age, y	52.8 ± 16.9	65.7 ± 13.6	<0.001	0.84
Male sex	155 (58.1)	51 (51.5)	0.263	0.13
Body mass index, kg/m^2^	29.2 ± 5.5	29.1 ± 5.1	0.882	0.02
Diabetes mellitus	115 (43.1)	62 (62.6)	<0.001	0.40
Current smoking	139 (52.1)	54 (54.5)	0.672	0.05
Peripheral artery disease	43 (16.1)	55 (55.6)	<0.001	0.90
Coronary artery disease	33 (12.4)	24 (24.2)	0.005	0.31
Prior myocardial infarction	11 (4.1)	7 (7.1)	0.246	0.13
Prior stroke	12 (4.5)	12 (12.1)	0.009	0.28
Heart failure	7/197 (3.6)	4/78 (5.1)	0.513	0.08
Average 24 h systolic BP, mm Hg	132.9 ± 16.9	144.1 ± 16.9	<0.001	0.66
Average 24 h diastolic BP, mm Hg	78.1 ± 11.9	76.8 ± 12.7	0.405	0.10
Lp(a), g/L ^†^	0.24 (0.08–0.77)	0.75 (0.10–1.03)	<0.001	0.47
Homocysteine, μmol/L ^†^	10.0 (8.0–15.5)	13.0 (9.9–16.9)	<0.001	0.45
High-sensitivity C-reactive protein, mg/L ^†^	3.00 (0.80–7.85)	3.10 (1.00–11.00)	0.113	0.19
Glycated haemoglobin, % ^†^	5.5 (5.0–6.1)	6.1 (5.3–7.4)	<0.001	0.42
Low-density lipoprotein cholesterol, mmol/L	2.9 ± 1.1	3.0 ± 1.4	0.285	0.13
High-density lipoprotein cholesterol, mmol/L	1.6 ± 0.8	1.7 ± 1.3	0.590	0.07
Total cholesterol, mmol/L	4.8 ± 1.5	5.1 ± 2.1	0.198	0.16
Triglycerides, mmol/L ^†^	0.38 (0.22–0.64)	0.40 (0.26–0.67)	0.485	0.07
Statin therapy	68 (25.5)	44 (44.4)	<0.001	0.40
Estimated glomerular filtration rate, mL/min/1.73 m^2^	80.4 ± 16.2	74.8 ± 19.4	0.011	0.31
Albumin–creatinine ratio, mg/mmol ^†^	2.30 (0.90–9.00)	5.90 (1.15–14.00)	0.006	0.28

High arterial stiffness was defined as cfPWV ≥ 10 m/s. Data are shown as *n* (%), mean ± SD, or median (IQR) for variables marked †. Absolute SMDs describe between-group imbalance. BP, blood pressure; cfPWV, carotid–femoral pulse-wave velocity; Lp(a), lipoprotein(a); SMD, standardised mean difference.

**Table 3:** Multivariable associations of lipoprotein(a), homocysteine, and peripheral artery disease with carotid–femoral pulse-wave velocity.

Model	Variable	Coefficient (95% CI), m/s	*p* Value
Adjusted model	Lp(a), per 0.1 g/L	0.14 (0.08 to 0.19)	<0.001
	Homocysteine, per 5 μmol/L	0.25 (0.01 to 0.49)	0.04
Adjusted model plus peripheral artery disease	Lp(a), per 0.1 g/L	0.13 (0.08 to 0.18)	<0.001
	Homocysteine, per 5 μmol/L	0.26 (0.02 to 0.49)	0.03
	Peripheral artery disease	0.75 (0.22 to 1.27)	0.006
Multiplicative interaction model plus peripheral artery disease	Centred Lp(a) × centred homocysteine interaction	0.01 (−0.03 to 0.06)	0.55

Coefficients represent adjusted differences in cfPWV, in m/s. The base model adjusted for age, sex, body mass index, 24 h systolic BP, diabetes mellitus, chronic kidney disease, smoking status, and low-density lipoprotein cholesterol; the PAD model additionally included PAD. The interaction term used centred Lp(a), per 0.1 g/L, and centred homocysteine, per 5 μmol/L. BP, blood pressure; CI, confidence interval; cfPWV, carotid–femoral pulse-wave velocity; Lp(a), lipoprotein(a); PAD, peripheral artery disease.

**Table 4:** Additive interaction and marginal structural model analyses.

Measure	Estimate (95% CI)	*p* Value
Lp(a)–homocysteine relative excess risk due to interaction	−0.48 (−1.72 to 0.45)	0.41
Lp(a)–homocysteine attributable proportion due to interaction	−0.35 (−1.37 to 0.30)	0.33
Lp(a)–homocysteine synergy index	0.43 (−1.14 to 2.61)	0.61
Lp(a) × homocysteine additive interaction term for continuous cfPWV	−0.49 (−1.85 to 0.87)	0.48
Marginal structural model interaction term for high cfPWV, Lp(a) × homocysteine	−0.20 (−0.50 to 0.11)	0.20

Values are estimates with 95% CIs. All interaction estimates refer to the joint Lp(a)–homocysteine exposure pattern. *p* values for the relative excess risk due to interaction, attributable proportion due to interaction, and synergy index are descriptive approximations derived from the corresponding 95% CIs. CI, confidence interval; cfPWV, carotid–femoral pulse-wave velocity; Lp(a), lipoprotein(a).

## Data Availability

The data that support the findings of this study are available from the corresponding author on reasonable request, subject to ethical approval and data-sharing agreements.

## Supplementary Materials

The following supporting information can be downloaded at https://www.mdpi.com/article/10.3390/jcm15155949/s1. Supplementary Table S1. STROBE checklist for reporting of this cross-sectional study. Supplementary Table S2. Sensitivity analyses for the association of lipoprotein(a) and homocysteine with carotid–femoral pulse-wave velocity. Supplementary Figure S1. Participant flow diagram. Derivation of the analytical cohort from consecutive adults with treated hypertension assessed at the Dobney Hypertension Centre and associated vascular service between February 2016 and October 2019. Participants were included if complete clinical, biochemical, medication, Lp(a), homocysteine, and cfPWV data were available. The final cohort included 366 participants: 267 with cfPWV < 10 m/s and 99 with cfPWV ≥ 10 m/s. BP, blood pressure; cfPWV, carotid–femoral pulse-wave velocity; Lp(a), lipoprotein(a). Supplementary Figure S2. Observed and expected prevalence of high arterial stiffness. Observed and expected prevalence of high cfPWV, defined as ≥10 m/s, across joint Lp(a)–homocysteine groups. Blue bars show observed prevalence, and orange bars show prevalence expected under additivity. cfPWV, carotid–femoral pulse-wave velocity; Hcy, homocysteine; Lp(a), lipoprotein(a).
