# Supplementary material for: Independent and Joint Associations of Lipoprotein(a) and Homocysteine with Carotid–Femoral Pulse-Wave Velocity in Adults with Treated Hypertension: A Single-Centre Cross-Sectional Study
Source: J Clin Med. 2026 Jul 30;15(15):5949. doi: 10.3390/jcm15155949 (PMC13467251; doi:10.3390/jcm15155949)
Supplement: Supplementary file 1 [file jcm-15-05949-s001.zip › jcm-4432687-supplementary.pdf]

Journal of Clinical Medicine

MDPI

Supplementary Table and Figure Legends

Table S1. STROBE checklist for reporting of this cross-sectional study.

STROBE Item	Recommendation	Reported Location in Manuscript
1(a)	Indicate the study design with a commonly used term in the title or abstract.	Title; Abstract; Methods
1(b)	Provide an informative and balanced summary of what was done and what was found.	Abstract
2	Explain the scientific background and rationale for the investigation.	Introduction
3	State specific objectives, including any prespecified hypotheses.	Introduction
4	Present key elements of study design early in the paper.	Methods, Study Design and Population
5	Describe the setting, locations, and relevant dates, including periods of recruitment, exposure, follow-up, and data collection.	Methods, Study Design and Population
6(a)	Give eligibility criteria and the sources and methods of participant selection.	Methods, Study Design and Population; Supplementary Figure S1
7	Clearly define all outcomes, exposures, predictors, potential confounders, and effect modifiers.	Methods, Clinical Assessment; Laboratory Measurements; Statistical Analysis
8	For each variable of interest, give sources of data and details of methods of assessment or measurement.	Methods, Clinical Assessment; cfPWV Measurement
9	Describe efforts to address potential sources of bias.	Methods; Discussion, Strengths and Limitations
10	Explain how the study size was arrived at.	Methods, Study Design and Population
11	Explain how quantitative variables were handled in the analyses.	Methods, Statistical Analysis
12(a)	Describe all statistical methods, including those used to control for confounding.	Methods, Statistical Analysis
12(b)	Describe any methods used to examine subgroups and interactions.	Methods, Statistical Analysis
12(c)	Explain how missing data were addressed.	Methods, Statistical Analysis
12(d)	Describe analytical methods taking account of sampling strategy, if applicable.	Not applicable; single-centre consecutive clinical cohort
12(e)	Describe any sensitivity analyses.	Methods, Statistical Analysis
13(a)	Report numbers of individuals at each stage of the study.	Results, Section 3.1; Supplementary Figure S1
13(b)	Give reasons for non-participation at each stage, if applicable.	Not applicable; retrospective consecutive clinical cohort
14(a)	Give characteristics of study participants.	Results; Table 1
14(b)	Indicate the number of participants with missing data for each variable of interest.	Methods, Statistical Analysis; Table footnotes, where applicable
15	Report numbers of outcome events or summary measures.	Results; Table 1; Supplementary Figure S1
16(a)	Give unadjusted and adjusted estimates and their precision.	Results; Tables 2–4; Supplementary Table S2
16(b)	Report category boundaries when continuous variables are categorised.	Methods, Statistical Analysis; Results; Figure legends
16(c)	Consider translating estimates of relative risk into absolute risk for a meaningful period, if relevant.	Not applicable; cross-sectional study of cfPWV
17	Report other analyses, including subgroup, interaction, and sensitivity analyses.	Results; Tables 3 and 4; Supplementary Table S2; Figures
18	Summarise key results with reference to study objectives.	Discussion

19	Discuss limitations, including sources of potential bias or imprecision.	Discussion, Strengths and Limitations
20	Give a cautious overall interpretation considering objectives, limitations, multiplicity, and evidence from similar studies.	Discussion
21	Discuss the generalisability of the study findings.	Methods, Study Design and Population; Discussion
22	Give the source of funding and the role of funders.	Funding statement
23	Report ethical approval and consent, where applicable.	Institutional Review Board Statement; Informed Consent Statement
24	Describe data availability.	Data Availability Statement
25	Disclose conflicts of interest.	Conflicts of Interest Statement

Completed STROBE checklist with corresponding manuscript locations. STROBE, Strengthening the Reporting of Observational Studies in Epidemiology.

Table S2. Sensitivity analyses for the association of lipoprotein(a) and homocysteine with carotid–femoral pulse-wave velocity.

Analysis	Parameter	Estimate (95% CI)	*p* Value
Primary robust linear regression	Lp(a), per 0.1 g/L	0.135 (0.084 to 0.186)	<0.001
	Homocysteine, per 5 µmol/L	0.247 (0.007 to 0.486)	0.043
PAD-adjusted robust linear regression	Lp(a), per 0.1 g/L	0.130 (0.079 to 0.180)	<0.001
	Homocysteine, per 5 µmol/L	0.257 (0.019 to 0.494)	0.034
	Peripheral artery disease	0.746 (0.219 to 1.273)	0.006
Sensitivity model additionally adjusted for statin therapy	Lp(a), per 0.1 g/L	0.126 (0.074 to 0.177)	<0.001
	Homocysteine, per 5 µmol/L	0.249 (0.009 to 0.488)	0.042
	Peripheral artery disease	0.772 (0.239 to 1.305)	0.005
	Statin therapy	0.438 (−0.037 to 0.912)	0.071
Sensitivity model additionally adjusted for documented cardiovascular comorbidity	Lp(a), per 0.1 g/L	0.128 (0.076 to 0.179)	<0.001
	Homocysteine, per 5 µmol/L	0.230 (−0.013 to 0.473)	0.064
	Peripheral artery disease	0.713 (0.177 to 1.250)	0.009
	Prior stroke	0.689 (−0.153 to 1.531)	0.109
	Coronary artery disease	0.398 (−0.230 to 1.027)	0.214
	Prior myocardial infarction	−0.075 (−1.131 to 0.981)	0.889
	Documented heart failure	0.505 (−0.713 to 1.723)	0.417
Sensitivity model with log-transformed biomarkers	log Lp(a)	0.511 (0.342 to 0.680)	<0.001
	log homocysteine	0.618 (0.052 to 1.184)	0.032
	Peripheral artery disease	0.861 (0.338 to 1.385)	0.001
	log Lp(a) × log homocysteine interaction	0.081 (−0.322 to 0.484)	0.694
Ordinary least-squares regression with HC3 robust standard errors	Lp(a), per 0.1 g/L	0.152 (0.096 to 0.209)	<0.001
	Homocysteine, per 5 µmol/L	0.322 (0.087 to 0.557)	0.007
	Peripheral artery disease	0.970 (0.294 to 1.646)	0.005
Median regression	Lp(a), per 0.1 g/L	0.101 (0.047 to 0.155)	<0.001
	Homocysteine, per 5 µmol/L	0.233 (−0.021 to 0.488)	0.072
	Peripheral artery disease	0.701 (0.136 to 1.266)	0.015
Sensitivity model additionally adjusted for log hs-CRP	Lp(a), per 0.1 g/L	0.144 (0.089 to 0.199)	<0.001
	Homocysteine, per 5 µmol/L	0.306 (0.059 to 0.553)	0.015
	Peripheral artery disease	0.739 (0.209 to 1.270)	0.006
	log hs-CRP	−0.123 (−0.301 to 0.056)	0.179
Functional-form comparison	Linear model: AIC/BIC/adjusted R^2	1604.59/1651.42/0.292	—
	Spline model: AIC/BIC/adjusted R^2	1600.96/1671.21/0.310	—
	Global comparison: spline vs. linear model	F = 2.53; df = 6	0.021

Estimates are regression coefficients for cfPWV, in m/s. Models are labelled by specification. The statin-adjusted model added recorded statin therapy; the documented cardiovascular-comorbidity model added prior stroke, coronary artery disease, prior myocardial infarction, and documented heart failure. In the documented cardiovascular-comorbidity-adjusted sensitivity model, missing heart-failure documentation (n = 91) was coded as no documented heart failure using deterministic documented-disease coding; this model therefore retained the full analytical cohort (n = 366). CI, confidence interval; cfPWV, carotid–femoral pulse-wave velocity; HC3, heteroscedasticity-consistent type 3; hs-CRP, high-sensitivity C-reactive protein; Lp(a), lipoprotein(a); PAD, peripheral artery disease.

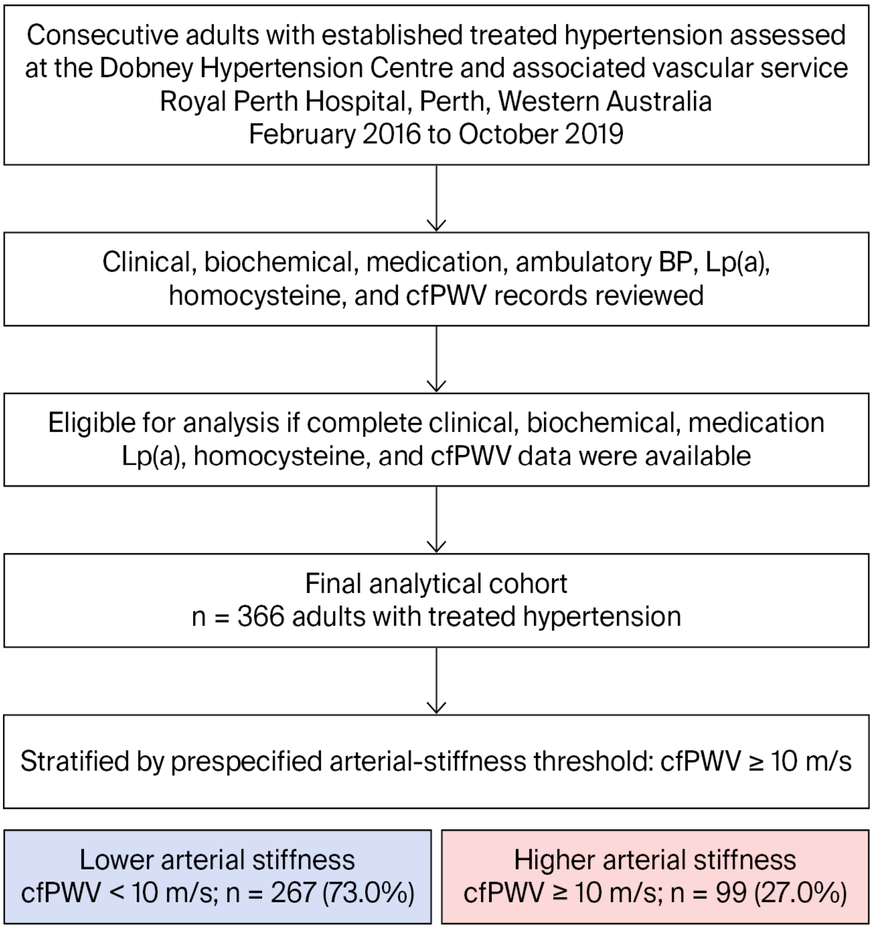

Figure S1. Participant flow diagram. *Derivation of the analytical cohort from consecutive adults with treated hypertension assessed at the Dobney Hypertension Centre and associated vascular service between February 2016 and October 2019. Participants were included if complete clinical, biochemical, medication, Lp(a), homocysteine, and cfPWV data were available. The final cohort included 366 participants: 267 with cfPWV <10 m/s and 99 with cfPWV ≥10 m/s. BP, blood pressure; cfPWV, carotid–femoral pulse-wave velocity; Lp(a), lipoprotein(a).*

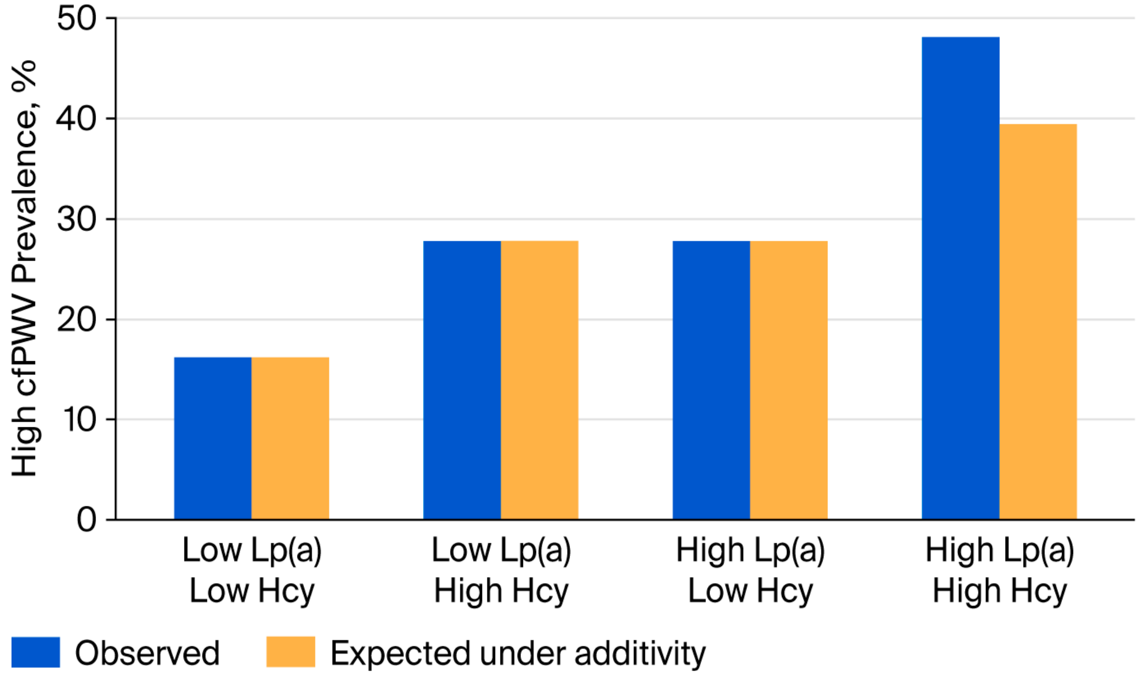

Figure S2. Observed and expected prevalence of high arterial stiffness. *Observed and expected prevalence of high cfPWV, defined as ≥10 m/s, across joint Lp(a)–homocysteine groups. Blue bars show observed prevalence, and orange bars show prevalence expected under additivity. cfPWV, carotid–femoral pulse-wave velocity; Hcy, homocysteine; Lp(a), lipoprotein(a).*
